# Skin Gene Expression Profiles in Systemic Sclerosis: From Clinical Stratification to Precision Medicine

**DOI:** 10.3390/ijms241612548

**Published:** 2023-08-08

**Authors:** Devis Benfaremo, Silvia Agarbati, Matteo Mozzicafreddo, Chiara Paolini, Silvia Svegliati, Gianluca Moroncini

**Affiliations:** 1 Department of Clinical and Molecular Sciences, Marche Polytechnic University, 60126 Ancona, Italy; d.benfaremo@staff.univpm.it (D.B.); s.agarbati@staff.univpm.it (S.A.); m.mozzicafreddo@staff.univpm.it (M.M.); c.paolini@staff.univpm.it (C.P.); s.svegliati@staff.univpm.it (S.S.); 2 Clinica Medica, Department of Internal Medicine, Marche University Hospital, 60126 Ancona, Italy

**Keywords:** systemic sclerosis, scleroderma, SSc, skin gene expression, fibrosis

## Abstract

Systemic sclerosis, also known as scleroderma or SSc, is a condition characterized by significant heterogeneity in clinical presentation, disease progression, and response to treatment. Consequently, the design of clinical trials to successfully identify effective therapeutic interventions poses a major challenge. Recent advancements in skin molecular profiling technologies and stratification techniques have enabled the identification of patient subgroups that may be relevant for personalized treatment approaches. This narrative review aims at providing an overview of the current status of skin gene expression analysis using computational biology approaches and highlights the benefits of stratifying patients upon their skin gene signatures. Such stratification has the potential to lead toward a precision medicine approach in the management of SSc.

## 1. Introduction

Cutaneous involvement is a hallmark of systemic sclerosis (SSc); it occurs in most patients and is the most accessible and obvious sign. The extent of skin involvement has been considered relevant for the prognosis of SSc since it is inversely correlated with survival and is considered an important marker of disease severity [1]. However, a recent EUSTAR study has questioned the prognostic value of conventional SSc classification into limited and diffuse skin subsets [2].

The skin encompasses three layers: epidermis, dermis, and subcutis (pannus, hypodermis), including skin appendages, vessels, and nerves. Skin involvement derives mainly from vasculopathy, from the inflammatory processes and appearance of myofibroblasts, and from the sclerotic processes in the dermis, which may be mutually influenced. The special interplay between cytokines and growth factors, which activate different mesenchymal cell populations and the production of extracellular matrix components, determines the biomechanical properties of the skin and the clinical features of systemic sclerosis [3].

Nearly all SSc patients develop skin involvement, notwithstanding the heterogeneity of both clinical and laboratory SSc manifestations. Skin involvement, especially sclerosis, which is the most characteristic cutaneous sign, is relevant for the classification and diagnosis of SSc.

In the 2013 classification criteria for systemic sclerosis, skin thickening of the fingers extending proximal to the metacarpophalangeal joints is sufficient for classifying the patient as having SSc [4].

Skin sclerosis is the visible result of histologically defined fibrosis of the skin, with excessive generation and closely packed collagen bundles, in which fibrils are probably cross-linked and arranged differently compared with healthy normal skin, accompanied by loss of vessels and sebaceous glands. The fibrotic process usually originates in the connective tissue of the septae in the subcutaneous layer (panniculus adiposus). Sclerosis, therefore, possesses a high specificity for SSc [5].

On the other hand, fibrosis or sclerosis are of low sensitivity when it comes to the early diagnosis of possible SSc. The earliest sign of skin involvement is usually Raynaud’s phenomenon, a sign of vasculopathy caused by vasoconstriction of cutaneous blood flow that occurs whenever there is cold exposure to the surface of the skin. Physiologically, in SSc patients, the endothelial environment is characterized by several alterations, including an imbalance between pro- and anti-angiogenic mediators, increased production of cell adhesion molecules, platelet activation, deregulation of vascular tonus, impaired compensatory vasculogenesis and angiogenesis, and production of reactive oxygen species (ROS) [6]. Morphological abnormalities in the peripheral microcirculation can be assessed by nailfold capillaroscopy. The characteristic “scleroderma pattern” comprises sequential and dynamic capillaroscopy results of widened capillaries, avascularity, hemorrhages, and distortion of the normal capillary architecture [7].

Swelling of the fingers (puffy hands) is another early cutaneous sign of SSc. The underlying edema may reflect pathologically increased vascular permeability. The VEDOSS (Very Early Diagnosis Of Systemic Sclerosis) study identified puffy fingers at the first visit as an independent parameter predicting the development of definite SSc [8]. Puffy hands, however, is a non-specific clinical sign of SSc, but it should be considered as a warning sign (a “red flag”), which should result in the follow-up of patients with this sign.

The direct and easy accessibility of the skin to inspection and palpation makes skin changes easily detectable and amenable to quantification. Sclerosis is characterized by a diffuse hardening and induration of the skin with loss of cutaneous elasticity and accompanying tightness. On palpation, the skin feels hard, and when trying to pinch it with the thumb and index finger, it feels thickened and difficult to be picked up due to the extension of fibrosis through the subcutis and the ensuing attachment of the dermis to the fascia. Sclerosis develops gradually in the course of the disease. It starts with edema, turns into sclerosis, and ends in an atrophic stage. In limited cutaneous SSc (lcSSc), in which sclerosis is confined to the extremities and face, it progresses slowly. In patients with dcSSc, more extensive skin thickening, extending beyond the extremities and face to include the complete limbs and trunk, characterizes the beginning of the disease.

Also, the physiological appearance of the skin may change with the simultaneous presence of hypo- and hyper-pigmentation in the same areas of the skin (‘salt and pepper’ skin) that yields a vitiligo-like look.

A frequent characteristic and very disabling complication of SSc is the occurrence of painful digital ulcers on the fingertips as a result of local ischemia and vascular insufficiency. Digital ulcers can be complicated by secondary bacterial infection, gangrene, and auto-amputation. They, therefore, need to be detected and treated early [9].

Also, the face becomes affected in SSc, developing characteristic features, including telangiectasias and microstomy. Further fibrosis leads to hardening of the facial skin so that the patient finds it increasingly difficult to exert mimic movements, and therefore, the face becomes expressionless, showing a mask-like stiffness.

SSc patients can also manifest calcinosis cutis due to the deposition of insoluble calcium salts in the skin and subcutaneous tissues. Recent studies support the vascular nature of calcinosis (more than an alteration in the calcium phosphate metabolism). This is also supported by evidence showing associations between calcinosis and advanced stages of microvascular changes on nailfold videocapillaroscopy [10].

An inability to extend or flex joints, especially of the fingers, is another important cutaneous manifestation of SSc. Tight fibrosis and ensuing stiffness lead to joint contracture.

Stiffness and deformity of the hands and changes of the face in SSc restrict patients’ everyday life, with considerable limitations in conducting common activities, taking care of themselves, coping with responsibility and stressful situations, maintaining personal relationships and friendships, and in work activities, forces many patients to quit their job, change their tasks, or face a decrease in their working productivity [11].

**Figure 1:**
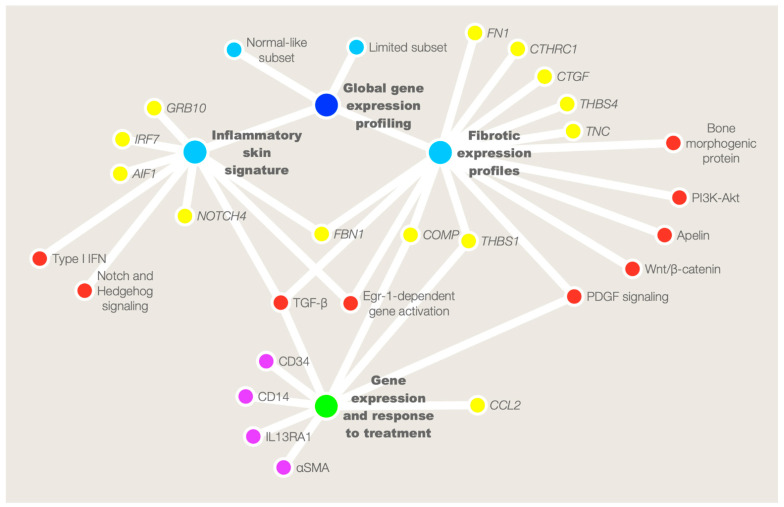
A representative network of skin gene expression profiles in systemic sclerosis, summarizing the most relevant genes and pathways resulting from literature revision. Global gene expression analysis (blue dot) revealed four intrinsic subsets (light blue dots) among patients with SSc: fibroproliferative, inflammatory, limited, and normal-like subsets. The present review focused on the inflammatory skin signature and the fibrotic expression profiles, highlighting their most relevant genes (yellow dots) and pathways (red dots). Moreover, gene expression and molecular signature in SSc may identify skin biomarkers (purple dots), genes, and pathways involved in the prediction of the trajectory of skin fibrosis and treatment response (green dot).

This review summarizes the current status of skin gene expression analysis using computational biology approaches (Figure 1) and emphasizes the benefits of stratifying patients upon their skin gene signatures.

## 2. Global Gene-Expression Profiling in SSc

SSc is a multifaceted disease that exhibits heterogeneity and clinical variability among patients, often complicating diagnosis and decisions regarding treatment. Gene expression profiling using microarray technology integrated with several bioinformatics techniques can provide information about the expression of thousands of genes in the human genome and identify pivot genes related to the pathology, as mentioned in a similar report by Keret et al. [12]. This approach has been successfully used in cancer and infectious disease research [13,14] and recently also in systemic autoimmune disorders [15,16].

The development of high-throughput sequencing technology and microarrays has been applied to systemic autoimmune rheumatic disease and, in particular, to a large cohort of SSc patients in order to provide a good opportunity to further understand this complex disease and overcome issues of clinical heterogeneity, increasing diagnostic accuracy and develop more effective treatments [15,17].

The wealth of data generated by genome-wide gene expression profiling on skin biopsies has allowed for the identification of patient subgroups contributing importantly to our understanding of pathogenesis and to molecular stratification of patients to categorize the heterogeneity in clinical presentations of scleroderma [18,19]. Genomic studies indicated that variability exists even within the two conventional SSc forms based on the extent of skin involvement. One of the first studies of gene expression heterogeneity in SSc identified four “intrinsic” gene expression subsets among patients with SSc. These included: (a) a fibroproliferative subset characterized by strong induction of proliferation genes; (b) a group characterized by robust upregulation of genes associated with both innate and adaptive immune responses; (c) a limited subset centered on a cluster of clinically limited patients; (d) a normal-like subset which consisted of both healthy controls and a subset of patients with both limited and diffuse SSc. This study provided a proof of concept that heterogeneity within SSc clinical groups could be measured using genome-wide molecular profiling [20].

Two subsequent cohorts of patients were studied to validate and expand upon the aforementioned investigations. Pendergrass et al. classified patients into fibroproliferative, inflammatory, and normal-like subsets based on predominant gene expression programs within each subset. He utilized serial biopsies to demonstrate that the abnormal gene expression patterns are established early in the disease and remain stable within patients, regardless of disease duration [21].

More recently, Assassi and colleagues conducted a comprehensive analysis of global gene expression profiles in a large sample of SSc patients and control subjects. They employed a comprehensive microarray platform to unravel the heterogeneity of transcriptome patterns in affected SSc skin. The majority of SSc patients exhibited distinct transcriptome profiles compared to control subjects. SSc skin samples prominently displayed fibroinflammatory and keratin transcript profiles. However, due to the coexistence of inflammatory and fibrotic signatures in most patients, they were unable to replicate the fibroproliferative subset identified in prior studies. A noteworthy finding from this study, shedding light on the etiology of the subsets, was the observation that patients categorized as normal-like had the longest disease duration, likely representing end-stage and inactive disease [22].

Recent studies have indicated that the histologic features of SSc lesional skin provide the most informative data regarding gene expression subsets. These features can be useful for guiding subsequent gene expression analyses. One study reported that two fibroblast markers (αSMA and CD34) strongly predict gene expression subsets and can assist in identifying patients who are more likely to show improvement [23].

In a study by Moon and colleagues, a methodology successfully employed in cancer and other infectious diseases was used to create the largest cohesive transcriptomics compendium for SSc. The goal was to understand differential expression patterns, identify key players and potential drug targets, as well as discover distinct patient clusters. Unbiased cluster analysis of the diffuse cutaneous SSc (dSSc) compendium allowed for the categorization of patients into subgroups based on pathway activation. This was achieved by utilizing literature data, functional gene-set enrichment analysis (GSEA), protein–protein interaction network analysis, and non-negative matrix factorization (NMF). Additionally, promising target pathways and biomarkers that correlated closely with the clinical index were identified [24].

Johnson et al. aimed to develop an efficient and powerful tool for creating gene signatures from SSc skin biopsies. They applied three well-established classification methods (SVM, RF, and NB) coupled with four different feature selections to clinically derived microarray data. Their classification method identified several genes that were not found using conventional differential gene analysis. It also demonstrated clear distinctions among patients based on skin scores, differentiating between high- and low-severity gene profiles [25].

The identification of difficult patient subsets has been recognized as a challenge in the success of various clinical trials and diagnostic purposes. Franks et al. addressed this issue by developing a method that utilizes reliable machine learning algorithms to allocate single samples to different gene expression subsets based on accurate and well-defined criteria. This method effectively assigns single samples to intrinsic gene expression subsets using objective molecular genomic data [26].

## 3. Inflammatory Skin Signature

Although inflammation plays a mechanistic role in the pathogenic events of SSc, the precise delineation of its components’ multiple functions remains unclear [27].

The utilization of RNA-Seq for genomic profiling of scleroderma skin is gaining traction as it enables the simultaneous characterization of a wide array of transcripts. This approach has the potential to reveal potential pathogenic pathways and determine associations between genetic signatures and clinical disease activity [28].

As mentioned earlier, gene expression signatures have currently defined four subsets in SSc: inflammatory, fibroproliferative, limited, and normal-like subsets [29].

The inflammatory subset is characterized by increased expression of genes associated with inflammation and extracellular matrix (ECM) deposition.

Mahoney et al. developed a computational tool to identify and characterize the biological role of consensus genes in SSc [30]. They identified an inflammatory-specific CC3 (community cluster) characterized by its enhanced response to interferons, B cell receptor signaling, TGFβ monocyte chemotaxis, PDGF signaling, and ECM remodeling processes. This inflammatory-specific consensus cluster includes genes such as *AIF1* and *FBN1*, the latter being implicated in SSc pathogenesis. Additionally, this consensus cluster contains polymorphic genes, like *NOTCH4*, *IRF7*, and *GRB10*, which consistently show differential expression in the inflammatory subset [30].

Skaug et al. investigated the transcript expression profiles of skin specimens from early systemic sclerosis patients. The results parallel the clinical observation that early SSc undergoes an inflammatory phase followed by a more fibrotic phase. Epigenetic changes occurring very early in the disease course, potentially years before fibrosis appears, may represent a plausible connection between inflammation and tissue fibrosis [31].

Furthermore, the results by Skaug and colleagues indicate that TGFβ, a key profibrotic cytokine implicated in the pathogenesis of SSc, seems to have a less prominent role in driving the dysregulated gene expression observed during this early inflammatory phase, contrary to its significant role in later-stage disease [32].

As previously mentioned, Moon and colleagues created a compendium of skin molecular signatures by analyzing transcriptomic data from patients with dSSc. This compendium further divided the inflammatory types into two subgroups: cluster 1 and cluster 2. Cluster 1 was found to be more strongly associated with Notch and Hedgehog signaling pathways, while cluster 2 exhibited a higher preference for the TGFβ signaling pathway. Both clusters were conceptually similar in terms of being inflammatory. Cluster 2, which had a shorter disease duration in the 24-week follow-up patients, was considered to represent the early inflammatory phase and demonstrated a more favorable clinical outcome due to its reversible immunoinflammatory nature, as opposed to fibrotic changes. Cluster 2 was also found to be highly enriched with immune cells such as B cells, T cells, dendritic cells, and macrophages, whereas cluster 1 exhibited a higher score for eosinophils and Th1 cells. Additionally, the study revealed that the type I IFN signaling pathway was more active in both clusters 1 and 2 [24].

More recently, the advent of single-cell RNA-sequencing technology allowed the characterization at the individual cellular level of SSc skin.

Werner et al. [33] described 12 diverse fibroblast subpopulations with an overall inflammatory gene signature in the affected skin of patients with localized scleroderma. Three subclusters, in particular, were more frequently distributed in SSc patients than in healthy controls, showing upregulation of genes involved in immune response (*CXCL9/10/11*- and *INF*-pathway, *HLA* expression), mesenchymal transition and wound healing (*SFRP2*, *PRSS23*, *SFRP4*, *ADAM12*, *ACTA2*, *MFAP5,* and *FBN1*), cell motility, proliferation, and apoptosis (*CXADR*, *GATA3*).

In a bleomycin-induced model of SSc skin, Chitturi and colleagues [34] identified, by spatial transcriptomics based on cellular location within the skin and cell cluster analysis, four cellular niches (epithelia, papillary, reticular. or universal fibroblasts) distinguished by gene expression of key markers. In particular, two different fibroblast populations were distinguished by the papillary/regenerative markers, Defb8 and Crabp1, and the reticular fibroblast markers Nexn, Actn2, Trim63, and Hspb7.

The deep characterization of unique SSc skin cellular populations provides new insight into tissue complexity and opens the way for the identification of novel potential cellular targets for personalized medicine.

## 4. Fibrotic Expression Profiles

Extracellular matrix (ECM) deposition plays a fundamental role in the initiation and progression of fibrotic mechanisms [35]. It involves various macromolecules such as glycoproteins, enzymes, and collagen [36]. In pathologies like SSc, characterized by an uncontrolled accumulation of ECM leading to non-functional fibrotic tissue, several biochemical signaling pathways are commonly involved. These pathways include the TGF-β cytokine, bone morphogenic protein (BMP), connective tissue growth factor (CTGF), Wnt/β-catenin, and platelet-derived growth factor (PDGF) [37,38].

Transcriptomic and gene expression analyses using microarray and Next Generation RNA-Sequencing (NGS) experiments have been performed on skin samples and monocyte-derived macrophages from SSc patients to identify dysregulated genes, both overexpressed and under-expressed [39]. The dysregulated genes, along with the overall activity of related compounds, contribute to the disease as a cumulative consequence of abnormal activation of common pathways [40].

Among the four clusters identified by Moon and colleagues, the fibroproliferative type, which showed the strongest inclination towards ECM formation, also exhibited high activity in TGF-β, Apelin, PI3K-Akt, and PDGF signaling pathways [24].

Using gene set enrichment analysis (GSEA), the PI3K-Akt pathway was classified as one of the most enriched gene sets in dSSc, and 94 genes were identified based on the leading edge of enrichment. Among the top 10 genes in this set were 5 genes related to the collagen family (*COMP*, *THBS1*, *THBS4*, *TNC*, and *FN1*) [24]. This finding has been confirmed by other studies, including Lofgren et al. [41], Pendergrass et al. [21], and Whitfield et al. [42].

Analyzing the results obtained with a microarray-based gene expression dataset from SSc skin biopsies, Bhattacharyya and colleagues observed an “Erg-1-regulated gene signature” clustered with the diffuse-proliferative SSc subset and not with the limited form or healthy controls [43]. At the genome-wide level, Erg-1, a transcription factor whose expression was found to be elevated in skin biopsies from dSSc patients, is involved in the modulation of genes implicated in ECM synthesis, proliferation, and wound healing. The identification of a distinct molecular subset of scleroderma distinguishable by “Erg-1-responsive gene signature” may be useful for a novel therapeutic strategy.

The analysis of protein–protein interaction networks identified 14 differentially expressed genes (DEGs) that had been previously identified as genetic susceptibility loci for SSc [44]. Among these DEGs, five genes were identified as potential target molecules for therapy [45]. Notably, CTGF, a secreted matricellular protein that contributes to tissue remodeling and fibrosis in cooperation with TGF-β, not only exhibited differential expression but also had a central hub status in the network [24,46].

Furthermore, Milano and colleagues reported that genes overexpressed in dSSc, lSSc, and morphea, which are related to the fibrosis process (including collagen genes, collagen triple helix repeat containing 1 (*CTHRC1*), and fibrillin-1 (*FBN1*)), showed co-expression with markers of macrophages and T lymphocytes [20].

## 5. Relationship of Gene Expression with Skin Trajectories and Response to Treatment

Gene expression and molecular signatures in SSc-derived skin biopsies might provide insights into the prediction of the trajectory of skin fibrosis and treatment response.

Several studies have examined the role of differential gene expression as a potential biomarker for disease progression. Skaug et al. demonstrated that immune cell signatures correlated with the rate of skin thickness progression before biopsy, but they did not predict subsequent progression of the modified Rodnan skin score (mRSS) [32]. Additionally, they found that skin gene expression tended to normalize over time. While immune cell and fibroblast gene expression signatures were predictive of longitudinal mRSS, they were not independently predictive when adjusting for baseline mRSS [47].

Lofgren et al. identified 415 differentially expressed genes (211 overexpressed, 204 underexpressed) as a signature in systemic sclerosis (SSc) patients compared to healthy controls. They established a disease severity measure called 4S [41]. The 4S significantly correlated with SSc severity, as measured by mRSS, across all skin biopsy datasets regardless of treatment, and it was unaffected by clinical factors such as obesity, edema, or experience in performing mRSS. Furthermore, changes in 4S between baseline and 12 months were significantly correlated with subsequent changes in skin disease severity up to 24 months. These findings suggest that gene signatures could be utilized to differentiate between treatment responders and non-responders before observable changes in mRSS, enabling earlier identification of treatment responders.

In a phase II study by Stifano and colleagues, which examined clinical data from 38 placebo-treated patients and gene expression from skin biopsies, skin biomarkers associated with macrophages (CD14, IL13RA1), and TGFβ activation (SERPINE1, CTGF, OSMR), were found to be prognostic for progressive disease in patients with dcSSc [48]. Specifically, patients in the highest tertile of CD14, IL13RA1, SERPINE1, OSMR, and CTGF expression showed an increased mRSS during follow-up compared to those in the lowest tertile.

In a recent study, two fibroblast markers (αSMA and CD34) emerged as the strongest predictors of gene expression subsets in dermal fibroblasts [23]. Specifically, samples with a high αSMA and low CD34 immunophenotype showed increased expression of several TGF-β-regulated genes. Notably, the expression of αSMA and collagen negatively correlated with clinical improvement, while CD34 expression positively correlated.

Although there may be limitations in using skin gene expression profiles to predict subsequent disease progression, possibly due to heterogeneity among systemic sclerosis (SSc) patient cohorts, it holds promise as a tool for patient stratification.

Interesting data are also being gathered regarding the role of gene expression signatures as biomarkers of treatment response.

Mycophenolate mofetil (MMF) is one of the most commonly used drugs for treating skin and lung disease in SSc. Previous studies by Hinchcliff and colleagues have demonstrated that a high baseline inflammatory gene expression signature in the skin of SSc patients was associated with significant improvement in the modified Rodnan skin score (mRSS) during MMF therapy [49]. Furthermore, discontinuation of MMF at 24 months resulted in an increased inflammatory score and levels of *CCL2* mRNA and protein, a rebound in mRSS, and an increase in the number of skin myeloid cells in SSc patients [50].

Tyrosine kinase inhibitors (TKIs) are drugs that target the kinases involved in the downstream pathway of various soluble mediators of inflammation and fibrosis. One such TKI, nintedanib, has recently been approved for the treatment of SSc-related interstitial lung disease based on favorable results from the SENSCIS and INBUILD studies [51,52]. Two other TKIs, imatinib and nilotinib, have also been studied for their effects on skin gene expression signatures.

Chung et al. conducted a study on two patients with dcSSc who responded to imatinib mesylate. Immunohistochemistry analysis of pre-treatment skin biopsy samples revealed high levels of phospho-PDGFR in dermal fibroblasts and phospho-Abl in vascular structures. Following initiation of imatinib therapy, there were reductions in both phospho-PDGFR and phospho-Abl. Gene expression profiling allowed the identification of an imatinib-responsive signature specific to dcSSc, involving genes associated with multiple functional pathways, including cell proliferation, matrix and vascular remodeling, immune signaling, and growth factor signaling [53].

In an open-label trial evaluating the use of nilotinib in SSc patients, Gordon and colleagues demonstrated that patients who showed improvement (defined as a decrease in mRSS > 20% from baseline at 12 months) had significantly higher expression of *TGFBR* and *PDGFRB* signaling genes compared to non-improvers. Furthermore, the expression of these genes significantly decreased in the improvers following treatment [54].

Collectively, these and other studies point to PDGFR as an amenable therapeutic target and biomarker of response to treatment in SSc [55].

The use of biologic drugs has gained attention in SSc, particularly after the FDA approval of tocilizumab (TCZ) for the treatment of interstitial lung disease associated with SSc. In the phase II faSScinate study, which investigated TCZ use in SSc patients, there was a trend toward benefits in terms of the primary endpoint, mRSS, and a strong trend at 48 weeks, along with consistent benefits in exploratory endpoints, including lung function [56]. Ancillary analyses demonstrated that the treatment of SSc patients with TCZ for 24 weeks tended to normalize the profibrotic expression profile of SSc dermal fibroblasts. This normalization was characterized by decreased levels of profibrotic proteins, reduced migration and contractility activities, and differential expression of a distinct set of fibrosis-related genes, with a high prevalence of TGF-β-regulated genes.

In a 52-week placebo-controlled trial that did not achieve its primary outcome, a significant decrease in the expression of B cell signaling and profibrotic genes and pathways was observed in patients with improved mRSS in the belimumab cohort but not in the placebo group [57]. Specifically, there were 76 significant differentially expressed genes (DEGs) whose levels decreased after treatment, encompassing immune (B cell) and fibrotic (TGF-β) signaling. There was no observed correlation between baseline intrinsic subsets and clinical response. However, improvers were more likely to be associated with a normal-like molecular subset following treatment, and a decrease in inflammatory gene expression signatures accompanied the decrease in mRSS.

In a randomized trial comparing abatacept vs. placebo, SSc patients with an inflammatory intrinsic subset showed a trend toward greater improvement in skin score at 24 weeks compared to patients in the normal-like intrinsic subset [58]. Additionally, while improvers showed decreased gene expression in inflammatory pathways over 24 weeks, non-improvers and placebo patients showed stable or reverse gene expression.

Among biological drugs targeting profibrotic pathways, fresolimumab, a neutralizing antibody that targets TGF-β, has been evaluated in an open-label trial in SSc patients [59]. The expression of the TGF-β-regulated biomarker genes thrombospondin-1 (*THBS1*) and cartilage oligomeric protein (*COMP*) were analyzed in serial mid-forearm skin biopsies obtained before and after treatment. Fresolimumab treatment resulted in a rapid decline in *COMP* and *THBS1* expression. Clinical improvement in skin involvement and a decrease in dermal myofibroblast infiltration were also observed after fresolimumab treatment. Additionally, baseline levels of *THBS1* were predictive of improved mRSS after treatment with fresolimumab.

JAK inhibitors are targeted therapies that inhibit the activity of Janus kinase family enzymes (JAK1, JAK2, JAK3, TYK2), thereby interfering with the JAK-STAT signaling pathway. Four JAK inhibitors are currently approved for the treatment of various inflammatory diseases, and tofacitinib is being evaluated for SSc. Khanna et al. conducted a study to evaluate changes in gene expression associated with tofacitinib treatment in different skin cell populations. They compared single-cell gene expression in punch skin biopsies obtained at baseline and 6 weeks after the initiation of treatment [60]. They found that fibroblast and keratinocyte subpopulations showed an interferon signature at baseline, but after treatment with tofacitinib, gene expression was downregulated in these cell subpopulations. However, no clinically significant inhibition of T cells and endothelial cells in the skin tissue was observed. As for tyrosine kinase 2 (TYK2), a significant association was reported between SSc and this gene and the related product IL-12 [61]. Thus, the use in SSc of selective TYK2 inhibitor deucravacitinib, currently approved for the treatment of psoriasis, may be conceivable.

More recently, Fukasawa and colleagues reported the effect of IL-23 and IL-17 inhibition in patients with SSc. In the first study [62], guselkumab was administered to three patients with concomitant psoriasis and SSc, showing a possible beneficial effect of IL-23 inhibition on immune abnormalities, fibrosis, and vascular damage. In the second phase I open-label study [63], all eight patients with early progressive SSc that received brodalumab, an Il-17 inhibitor, showed a decrease in mRSS that exceeded the minimal clinically important difference, with a significant reduction in dermal thickness.

## 6. Conclusions

Whereas SSc classification based on skin involvement extension has limited predictive value [2], gene expression and molecular signatures obtained from SSc-derived skin biopsies have the potential to provide valuable insights into predicting the progression of skin fibrosis and the response to treatment. By analyzing the gene expression patterns in these biopsies, researchers can identify specific molecular signatures associated with disease progression and treatment outcomes. These signatures can help in determining the trajectory of skin fibrosis, allowing for early intervention and personalized treatment strategies. Additionally, understanding the gene expression profiles can shed light on the underlying molecular mechanisms driving fibrosis in SSc, leading to the identification of potential therapeutic targets.

## 7. Future Directions

Overall, gene expression and molecular signatures derived from SSc skin biopsies hold promise for improving the prediction of skin fibrosis progression and treatment response in SSc patients. It should be emphasized that, in light of the heterogeneity of the patient populations and the inclusion criteria used, the results of these studies should be interpreted with caution. Indeed, stratification based on skin induration alone is not sufficient to capture the full spectrum of SSc disease [2]. Recently, other data from the EUSTAR cohort demonstrated that patients with sine scleroderma SSc may have as high a disease burden as patients with skin involvement [64]. Further studies are required to enhance our understanding of how gene expression profiles in the skin can be effectively translated into clinical practice, ultimately leading to improved management of SSc patients.

## Data Availability

Data sharing is not applicable to this article as no new data were created or analyzed in this study.
